# Association Between Immune-Related Adverse Events and Survival in Patients with Hepatocellular Carcinoma Treated With Atezolizumab Plus Bevacizumab

**DOI:** 10.1093/oncolo/oyad090

**Published:** 2023-04-06

**Authors:** Taito Fukushima, Manabu Morimoto, Satoshi Kobayashi, Makoto Ueno, Haruki Uojima, Hisashi Hidaka, Chika Kusano, Makoto Chuma, Kazushi Numata, Kota Tsuruya, Yoshitaka Arase, Tatehiro Kagawa, Nobuhiro Hattori, Hiroki Ikeda, Tsunamasa Watanabe, Katsuaki Tanaka, Shin Maeda

**Affiliations:** Division of Hepatobiliary and Pancreatic Oncology, Kanagawa Cancer Center, Yokohama, Japan; Division of Hepatobiliary and Pancreatic Oncology, Kanagawa Cancer Center, Yokohama, Japan; Division of Hepatobiliary and Pancreatic Oncology, Kanagawa Cancer Center, Yokohama, Japan; Division of Hepatobiliary and Pancreatic Oncology, Kanagawa Cancer Center, Yokohama, Japan; Department of Gastroenterology, Internal Medicine, Kitasato University School of Medicine, Sagamihara, Japan; Department of Gastroenterology, Internal Medicine, Kitasato University School of Medicine, Sagamihara, Japan; Department of Gastroenterology, Internal Medicine, Kitasato University School of Medicine, Sagamihara, Japan; Gastroenterological Center, Yokohama City University Medical Center, Yokohama, Japan; Gastroenterological Center, Yokohama City University Medical Center, Yokohama, Japan; Division of Gastroenterology and Hepatology, Department of Internal Medicine, Tokai University School of Medicine, Isehara, Japan; Division of Gastroenterology and Hepatology, Department of Internal Medicine, Tokai University School of Medicine, Isehara, Japan; Division of Gastroenterology and Hepatology, Department of Internal Medicine, Tokai University School of Medicine, Isehara, Japan; Division of Gastroenterology and Hepatology, Department of Internal Medicine, St. Marianna University School of Medicine, Kawasaki, Japan; Division of Gastroenterology and Hepatology, Department of Internal Medicine, St. Marianna University School of Medicine, Kawasaki, Japan; Division of Gastroenterology and Hepatology, Department of Internal Medicine, St. Marianna University School of Medicine, Kawasaki, Japan; Gastroenterology Division, Hadano Red Cross Hospital, Hadano, Japan; Department of Gastroenterology, Yokohama City University Graduate School of Medicine, Yokohama, Japan

**Keywords:** hepatocellular carcinoma, immune-related adverse events, Atezolizumab plus bevacizumab, landmark analysis, immunotherapy

## Abstract

**Background:**

Immune checkpoint inhibitors (ICIs) are effective for advanced hepatocellular carcinoma (HCC). However, there are few reports on the correlation between the clinical efficacy of ICIs and the development of immune-related adverse events (irAEs) in patients with HCC. The aim of this study was to investigate the association between irAE development and survival in patients with HCC treated with atezolizumab plus bevacizumab.

**Patients and Methods:**

We enrolled 150 patients with advanced HCC treated with atezolizumab plus bevacizumab between October 2020 and October 2021 at 5 territorial institutions. We compared the efficacy of atezolizumab plus bevacizumab between patients who experienced irAEs (irAE group) and those who did not (non-irAE group).

**Results:**

Thirty-two patients (21.3%) developed irAEs of any grade. Grade 3/4 irAEs were observed in 9 patients (6.0%). The median progression-free survivals (PFS) in the irAE and non-irAE groups were 273 and 189 days, respectively (*P* = .055). The median overall survivals (OS) in the irAE and non-irAE groups were not reached and 458 days, respectively (*P* = .036). Grade 1/2 irAEs significantly prolonged PFS (*P* = .014) and OS (*P* = .003). Grade 1/2 irAEs were significantly associated with PFS (hazard ratio [HR], 0.339; 95% confidence interval [CI], 0.166-0.691; *P* = .003) and OS (HR, 0.086; 95% CI, 0.012-0.641; *P* = .017) on multivariate analysis.

**Conclusion:**

The development of irAEs was associated with increased survival in a real-world population of patients with advanced HCC treated with atezolizumab plus bevacizumab. Grade 1/2 irAEs were strongly correlated with PFS and OS.

## Teaser

Immune checkpoint inhibitors (ICIs) are effective for advanced hepatocellular carcinoma (HCC), but the correlation between the clinical efficacy of ICIs and the development of immune-related adverse events (irAEs) in patients with HCC is unclear. This article reports on the association between irAE development and survival in patients with HCC treated with atezolizumab plus bevacizumab.

**Implications for Practice**

The occurrence of irAEs is considered a biomarker for predicting the efficacy of ICIs. However, little is known about the relationship between the development of irAEs and the efficacy of ICIs in patients with HCC. This study revealed that the development of irAEs was associated with increased survival in patients with HCC treated with atezolizumab plus bevacizumab. In addition, grade 1/2 irAEs were strongly correlated with PFS and OS. Therefore, the development of irAEs predicts ICI efficacy, which suggests that cautious management of irAEs can lead to clinical benefit.

## Introduction

Hepatocellular carcinoma (HCC) is the sixth most common cancer and the third leading cause of cancer-related death worldwide.^1^ Early diagnosis of HCC is difficult, and one-third of cases are diagnosed at an advanced stage due to delayed diagnosis.^2,3^ Although for many years sorafenib was the only available systemic therapy for advanced HCC, treatment options have since expanded. Immune checkpoint inhibitors (ICIs) have recently been found to be effective in treating advanced HCC. Although ICI monotherapy as first- and second-line treatment failed to significantly prolong overall survival (OS) in a phase III study, it showed promising clinical activity.^4,5^ Further, in the HIMALAYA phase III study, a single priming dose of tremelimumab plus once-monthly durvalumab improved survival compared to sorafenib monotherapy.^6^ In the IMbrave 150 phase III study, atezolizumab plus bevacizumab resulted in better survival than sorafenib alone. As a result, this regimen was positioned as the standard therapy for the front-line treatment of advanced HCC.^7^

Although ICIs show favorable therapeutic effects, they often induce immune-related adverse events (irAEs). IrAEs are adverse events that are specific to ICIs and are different from the toxicities usually experienced with conventional systemic chemotherapy. IrAEs are inflammatory reactions with a unique spectrum that happen as a consequence of the aberrant overactivation of the immune system by ICIs. They can affect various organs, including the skin, gastrointestinal, respiratory, thyroid, and pituitary glands, and occur at any time, and persist even after treatment is discontinued. IrAEs often require steroid treatment to relieve symptoms, and in severe cases, ICI treatment must be discontinued, and a second line of immunosuppression agent must be added.^8^ Several recent studies have shown that irAEs are associated with the efficacy of ICIs in patients with melanoma and non-small cell lung cancer.^9-13^ In addition, similar studies have been reported for other malignancies, such as renal cell carcinoma, bladder carcinoma, and gastric cancer.^14-16^ However, little is known about the relationship between the development of irAEs and the prognosis of patients with HCC.^17-20^

Therefore, we performed a multicenter retrospective study to investigate the profile of irAEs and their association with survival outcomes in a real-world population of HCC patients treated with atezolizumab plus bevacizumab.

## Materials and Methods

### Patients

This retrospective study included 150 consecutive patients with unresectable HCC treated with atezolizumab plus bevacizumab between October 1, 2020 and October 30, 2021 at 5 institutions in the Kanagawa Liver Study Group: the Kanagawa Cancer Center, Kitasato University Hospital, Yokohama City University Medical Center, Tokai University Hospital, and St. Marianna University School of Medicine Hospital. Clinical data regarding patient characteristics, profile of irAEs, tumor response, and survival outcomes were obtained from the medical records. The data cutoff date was April 1, 2022. The study protocol was approved by the institutional review board of each participating center and was conducted in accordance with the Declaration of Helsinki (as revised in Fortaleza, Brazil, October 2013). The institutional review board waived the requirement for written informed consent due to the retrospective nature of this study. All patients were provided an opportunity to opt out of the study.

### Treatment and Assessment

Atezolizumab plus bevacizumab (1,200 mg of atezolizumab plus 15 mg/kg body weight of bevacizumab) was administered intravenously every 3 weeks. Treatment was continued until tumor progression or development of unmanageable adverse events. IrAEs were defined as adverse effects with a potential immunological basis that required more frequent monitoring and potential intervention with immune suppression or endocrine therapy. These were graded according to the National Cancer Institute Common Terminology Criteria for Adverse Events (version 5.0). We compared the efficacy of atezolizumab plus bevacizumab between patients who experienced irAEs (irAE group) and those who did not (non-irAE group). Tumor response was assessed using CT or MRI at baseline and every 6-9 weeks during treatment or whenever there was suspicion of disease progression. Radiological assessments were determined according to the Response Evaluation Criteria in Solid Tumours (RECIST) 1.1.^21^ Progression-free survival (PFS) was defined as the period from the date of atezolizumab plus bevacizumab initiation to the date of disease progression or death. OS was defined as the period from the date of atezolizumab plus bevacizumab initiation to death from any cause.

### Statistical Analyses

Continuous variables were compared using the Mann-Whitney U test, and categorical variables were compared using Fisher’s exact test or the χ^2^ test. Statistical significance was set at *P* < .05. Survival probabilities were estimated using the Kaplan-Meier method and compared using the log-rank test. To consider the lead-time bias due to the time-dependent nature of irAEs, landmark analysis was performed including only patients who had disease control or were alive at 9 weeks after starting atezolizumab plus bevacizumab. Multivariate analyses using a Cox proportional hazards regression model were performed to explore prognostic factors for OS and PFS. We selected 5 covariate factors (Eastern Cooperative Oncology Group status, Child-Pugh class, macrovascular invasion, alpha-fetoprotein, and line of therapy) that were considered clinically significant in terms of OS and PFS. Statistical analyses were performed using SPSS version 25 for Windows (IBM Corp., Armonk, NY, USA).

## Results

### Clinical Characteristics

Patient characteristics are summarized in Table 1. The median patient age was 72 years, and 120 patients (80.0%) were men. There were 54 (36.0%) and 96 (64.0%) patients with Barcelona Clinic Liver Cancer stages B and C, respectively. Thirty-three patients (22.0%) had macroscopic vascular invasion, and 58 patients (38.7%) had extrahepatic metastasis. A total of 132 patients (88.0%) had Child-Pugh class A liver function. Eighty-eight patients (58.7%) received atezolizumab plus bevacizumab as first-line treatment. There were no significant differences in clinicopathological characteristics between the irAE and non-irAE groups.

**Table 1.** Baseline characteristics.

	All patients *n* = 150	irAE group *n* = 32	Non-irAE group *n* = 118	*P*
Age, years	72 (65.0-77.0)	73.5 (68.3-76.7)	72 (65.0-76.8)	.243
Sex				.765
Male	120	25	95	
Female	30	7	23	
BMI, kg/m^2^	23.9 (21.1-25.9)	22.9 (21.4-26.0)	24.2 (21.1-26.0)	.324
Etiology				.291
HCV	49	14	35	
HBV	27	4	23	
Nonviral	74	14	60	
ECOG performance status				.052
0	117	20	97	
1	31	11	20	
2	2	1	1	
BCLC stage				.144
B	54	8	46	
C	96	24	72	
Macrovascular invasion				.644
No	117	24	93	
Yes	33	8	25	
Extrahepatic metastasis				.506
No	92	18	74	
Yes	58	14	44	
Child‒Pugh class				.082
A	132	31	101	
B	18	1	17	
ALBI grade				.696
Grade 1	53	13	40	
Grade 2	95	19	76	
Grade 3	2	0	2	
Albumin, g/dL	3.7 (3.4-4.0)	3.8 (3.5-4.0)	3.7 (3.4-4.0)	.249
Total bilirubin, mg/dL	0.8 (0.6-1.1)	0.7 (0.5-0.9)	0.8 (0.6-1.2)	.228
AFP, ng/mL	117.3 (7.5-1030.5)	42.7 (5.0-692.0)	135.0 (8.0-1559.0)	.172
CRP, ng/mL	0.4 (0.2-1.2)	0.3 (0.1-0.9)	0.5 (0.2-1.4)	.108
Line of therapy				.212
First line	88	23	65	
Second line	44	7	37	
≥Third line	18	2	16	

Values are presented as *n* or median (IQR).

Abbreviations: irAE, immune-related adverse event; IQR, interquartile range [25th-75^t^h percentile]; BMI, body mass index; ECOG, Eastern Cooperative Oncology Group; HCV, hepatitis C virus; HBV, hepatitis B virus; BCLC, Barcelona Clinic Liver Cancer; ALBI, albumin-bilirubin; AFP, alpha-fetoprotein; CRP, C-reactive protein.

### irAE Profiles

The irAE profiles are summarized in Table 2. Overall, 32 patients (21.3%) developed irAEs of any grade, with a median time to onset of 111 days. The most common irAEs were endocrine disorders (*n* = 11, 7.3%) and dermatological disorders (*n* = 9, 6.0%). Endocrine irAEs included thyroid dysfunction (*n* = 7, 4.7%), adrenal dysfunction (*n* = 3, 2.0%), and pituitary dysfunction (*n* = 1, 0.7%). Grade 3/4 irAEs were observed in 9 patients (6.0%), including endocrine (*n* = 3, 2.0%), gastrointestinal (*n* = 1, 0.7%), hepatic (*n* = 1, 0.7%), hematological (*n* = 1, 0.7%), pulmonary (*n* = 1, 0.7%), cardiovascular (*n* = 1, 0.7%), and nervous system (*n* = 1, 0.7%) irAEs. Overall, 26 patients (17.3%) experienced a single irAE and 6 patients (4.0%) experienced multiple irAEs. Twenty-three patients (71.7%) required treatment interruption, 13 patients (40.6%) permanently discontinued treatment, and 22 patients (68.8%) required systemic steroid treatment. Among 23 patients with grade 1/2 irAEs, 14 (61.0%) required treatment interruption, 5 (21.7%) permanently discontinued treatment, and 13 (56.5%) required systemic steroid treatment. The reasons for permanent discontinuation after grade 1/2 irAEs were based on the decision of the attending physicians considering the patient’s condition (*n* = 4) and disease progression during treatment interruption (*n* = 1). Among 9 patients with grade 3/4 irAEs, all (100%) required treatment interruption, 8 (88.9%) permanently discontinued treatment, and all (100%) required systemic steroid treatment.

**Table 2.** Profile of irAEs.

	Patients, *n* (%)			Median days to onset	Treatment interruption, *n*	Permanent treatment discontinuation, *n*	Received systemic steroid treatment, *n*
	Total	Grade 1/2	Grade 3/4				
Any irAE	32 (21.3)	23 (15.3)	9 (6.0)	111	23	13	22
Endocrine	11 (7.3)	8 (5.3)	3 (2.0)	133	4	4	6
Dermatologic	9 (6.0)	9 (6.0)	0 (0)	168	6	3	5
Gastrointestinal	4 (2.7)	3 (2.0)	1 (0.7)	124	3	0	4
Hepatic	3 (2.0)	2 (1.3)	1 (0.7)	96	3	1	2
Hematological	3 (2.0)	2 (1.3)	1 (0.7)	125	2	1	2
Pulmonary	2 (1.3)	1 (0.7)	1 (0.7)	154.5	2	2	2
Musculoskeletal	2 (1.3)	2 (1.3)	0 (0)	96.5	2	1	1
Cardiovascular^*^	1 (0.7)	0 (0)	1 (0.7)	8	1	1	1
Nervous system	1 (0.7)	0 (0)	1 (0.7)	7	1	1	1
Renal	1 (0.7)	1 (0.7)	0 (0)	103	1	0	1

^*^This case was reported by Isasaki et al.^22^

Abbreviations: irAE, immune-related adverse event.

### Efficacy analysis

As of the clinical data cutoff date, the median follow-up was 282.5 days. During the follow-up period, 91 patients had disease progression and 51 patients died. Table 3 shows the radiological assessments according to the RECIST criteria. The objective response rates (ORRs) were 37.5% in the irAE group and 24.6% in the non-irAE group (*P* = .146). The disease control rates (DCRs) were 84.4% in the irAE group and 73.7% in the non-irAE group (*P* = .211).

**Table 3.** Radiological assessment using RECIST 1.1.

	irAE group, *n* (%) *n* = 32	Non-irAE group, *n* (%) *n* = 118	*P*
CR	1 (3.1)	0 (0)	
PR	11 (34.4)	29 (24.6)	
SD	15 (46.9)	58 (49.2)	
PD	3 (9.4)	27 (22.9)	
NE	2 (6.3)	4 (3.4)	
ORR (CR+PR)	12 (37.5)	29 (24.6)	.146
DCR (CR+PR+SD)	27 (84.4)	87 (73.7)	.211

Abbreviations: RECIST, Response Evaluation Criteria in Solid Tumours; irAE, immune-related adverse event; CR, complete response; PR, partial response; SD, stable disease; PD, progressive disease; NE, not evaluated; ORR, overall response rate; DCR, disease control rate.

We further analyzed the radiological assessments according to the RECIST criteria based on the severity of irAEs. The ORRs were 34.8% among patients with grade 1/2 irAEs and 44.4% among those with grade 3/4 irAEs (*P* = .612). The DCRs were 91.3% among patients with grade 1/2 irAEs and 66.7% among patients with grade 3/4 irAEs (*P* = .084). There were no significant differences in the ORR or DCR between patients with grade 1/2 and grade 3/4 irAEs or between patients with grade 1/2 irAEs and those without irAEs.

**Figure 1.**
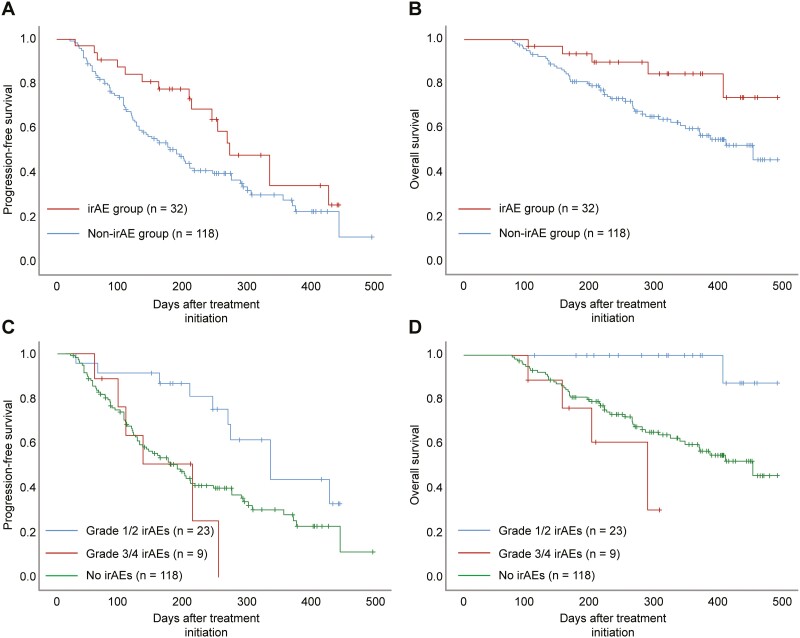
Kaplan-Meier survival estimates of PFS and OS. (**a**) PFS in patients with and without irAEs (*P* = .055). (**b**) OS in patients with and without irAEs (*P* = .036). (**c**) PFS based on irAE grade (grade 1/2 vs grade 3/4: *P* = .002; grade 1/2 irAEs vs no irAEs: *P* = .014). (**d**) OS based on irAE grade (grade 1/2 vs grade 3/4: *P* < .001; grade 1/2 irAEs vs no irAEs: *P* = .003). PFS, progression-free survival; OS, overall survival; irAE, immune-related adverse event.

The Kaplan-Meier curves for PFS and OS are shown in Fig. 1. The median PFS were 273 days (95% CI, 202.7-343.3) in the irAE group and 189 days (95% CI, 144.2-233.8) in the non-irAE group (*P* = .055). The median PFS were 336 days (95% CI, 241.2-430.8) among patients with grade 1/2 irAEs and 213 days (95% CI, 104.9-321.1) among patients with grade 3/4 irAEs (*P* = .002). Patients with grade 1/2 irAEs had better PFS than patients with grade 3/4 irAEs and those without irAEs (*P* = .014). The median OS were not reached (95% CI, not reached) in the irAE group and 458 days (95% CI, not reached) in the non-irAE group (*P* = .036). The median OS was not reached (95% CI, not reached) among patients with grade 1/2 irAEs and 292 days (95% CI, 158.3-425.7) among patients with grade 3/4 irAEs (P < .001). Patients with grade 1/2 irAEs had significantly better OS than patients with grade 3/4 irAEs and those without irAEs (*P* = .003). Further, multivariate analysis revealed that grade 1/2 irAEs were significantly associated with increased PFS (HR, 0.339; 95% CI, 0.166-0.691; *P* = .003) and OS (HR, 0.086; 95% CI, 0.012-0.641; *P* = .017) (Table 4).

**Table 4.** Multivariate analysis of factors associated with PFS or OS.

	PFS		OS	
	HR (95% CI)	*P*	HR (95% CI)	*P*
irAEs (grade 1/2 vs. grade 3/4 or no irAEs)	0.339 (0.166-0.691)	.003	0.086 (0.012-0.641)	.017
ECOG status (0 vs. 1 or 2)	0.536 (0.320-0.897)	.018	0.733 (0.376-1.429)	.362
Child-Pugh class (A vs. B)	0.471 (0.252-0.880)	.018	0.489 (0.234-1.024)	.058
Macrovascular invasion (yes vs. no)	2.093 (1.269-3.452)	.004	1.895 (0.947-3.792)	.071
AFP (<400 ng/mL vs. ≥400 ng/mL)	0.619 (0.397–0.966)	.034	0.516 (0.289-0.921)	.025
Line of therapy (first-line vs. ≥second-line)	0.618 (0.401-0.951)	.029	0.690 (0.378–1.260)	.227

Abbreviations: irAE, immune-related adverse event; HR, hazard ratio; CI, confidence interval; ECOG, Eastern Cooperative Oncology Group; OS, overall survival; PFS, progression-free survival; AFP, alpha-fetoprotein.

We further analyzed PFS and OS based on irAE category. The median PFS and OS for patients with endocrine irAEs were not reached (95% CI, not reached). Further, patients with endocrine irAEs had significantly longer PFS (*P* = .008) and OS (*P* = .031) than patients without endocrine irAEs. Dermatological, gastrointestinal, hepatic, and hematological irAEs were not associated with PFS or OS. Other irAEs were excluded from the analysis due to the small sample size.

### Landmark analysis

A total of 127 patients (84.7%) were included in the 9-week landmark analysis. The median PFS were 336 days (95% CI, 259.6-412.4) in the irAE group and 217 days (95% CI, 147.1-286.9) in the non-irAE group (*P* = .154). The median PFS were 336 days (95% CI, 193.0-479.0) among patients with grade 1/2 irAEs and 213 days (95% CI, 93.9-332.1) among patients with grade 3/4 irAEs (*P* = .002). Patients with grade 1/2 irAEs had significantly better PFS than patients with grade 3/4 irAEs and those without irAEs (*P* = .043). The median OS were not reached (95% CI, not reached) in the irAE group and 458 days (95% CI, not reached) in the non-irAE group (*P* = .073). The median OS were not reached (95% CI, not reached) among patients with grade 1/2 irAEs and 292 days (95% CI, 159.9-424.4) among patients with grade 3/4 irAEs (P < .001). Patients with grade 1/2 irAEs had significantly better OS than patients with grade 3/4 irAEs and those without irAEs (*P* = .010). Multivariate analysis revealed that grade 1/2 irAEs were significantly associated with increased PFS (HR, 0.312; 95% CI, 0.143-0.680; *P* = .003) and OS (HR, 0.081; 95% CI, 0.011-0.614; *P* = .015) (Table 5).

**Table 5.** Multivariate analysis of factors associated with PFS or OS (landmark analysis).

	PFS		OS	
	HR (95% CI)	*P*	HR (95% CI)	*P*
irAEs (grade 1/2 vs. grade 3/4 or no irAEs)	0.312 (0.143-0.680)	.003	0.081 (0.011-0.614)	.015
ECOG status (0 vs. 1 or 2)	0.448 (0.246-0.816)	.009	0.461 (0.216-0.981)	.044
Child-Pugh class (A vs. B)	0.479 (0.218–1.052)	.067	0.433 (0.170-1.103)	.079
Macrovascular invasion (yes vs. no)	2.537 (1.418-4.538)	.002	2.112 (0.937-4.759)	.071
AFP (<400 ng/mL vs. ≥400 ng/mL)	0.522 (0.313-0.870)	.013	0.422 (0.218–0.818)	.011
Line of therapy (first-line vs. ≥second-line)	0.669 (0.409-1.094)	.109	0.787 (0.394-1.572)	.498

Abbreviations: irAE, immune-related adverse event; OS, overall survival; PFS, progression-free survival; HR, hazard ratio; CI, confidence interval; ECOG, Eastern Cooperative Oncology Group; AFP, alpha-fetoprotein.

## Discussion

In this study, we investigated irAE profiles and the correlation between irAEs and clinical efficacy in a real-life HCC population treated with atezolizumab plus bevacizumab. It has been previously reported that the occurrence of irAEs after ICI treatment in patients with HCC is associated with prolonged outcome.^17-20^ However, no previous reports were limited to atezolizumab plus bevacizumab, and all included patients treated with single agents. Furthermore, there have been no reports focusing on the irAEs associated with atezolizumab plus bevacizumab in other malignancies. To our knowledge, this is the first study to report the irAE profiles associated with atezolizumab plus bevacizumab and their association with survival outcomes.

The proportion of patients who experienced irAEs in this study (21.3%) was similar to that in previous studies on HCC (7.7%–20.8%).^17-20^ The IMbrave150 trial, which examined atezolizumab plus bevacizumab, reported that 68.7% of patients developed atezolizumab-related adverse events.^7^ However, that study included adverse events other than irAEs. In particular, the frequency of hepatitis was markedly different from that in our study; 43.2% of patients in the Imbrave 150 study developed hepatitis, whereas only 2.0% of our patients developed hepatitis. Hepatitis could be disease-related or caused by concomitant drug effects (including alcohol) or infection, which could be one reason for this difference in results. The KEYNOTE-240 trial treating patients with pembrolizumab reported an irAE frequency of 18.3%, which was comparable to our results.^5^ Therefore, the incidence of irAEs in this real-world population was similar to that seen in clinical trials.

This study revealed that the irAE group had a significantly longer OS than the non-irAE group. Furthermore, the irAE group had a higher ORR and prolonged PFS compared to the non-irAE group, although there were no significant differences. Our results are consistent with those of previous studies in patients with other malignancies that have reported a potential association between the occurrence of irAEs and treatment efficacy. The significant difference in OS, despite the lack of significant difference in PFS, may be largely due to the characteristics of HCC. As 5 tyrosine kinase inhibitors (sorafenib, lenvatinib, regorafenib, cabozantinib, and ramucirumab) are currently available in Japan for patients who experience disease progression after atezolizumab plus bevacizumab, sequential treatment may have affected OS.^23-27^ Terashima et al revealed that the correlation between OS and post-progression survival was strong, whereas that between OS and time to progression was weak.^28^ Therefore, sequential therapy after atezolizumab plus bevacizumab in this study may have prolonged OS by prolonging post-progression survival.

Notably, our results showed that grade 1/2 irAEs significantly prolonged PFS and OS compared to not only grade 3/4 irAEs but also no irAEs. Moreover, multivariate analysis revealed that grade 1/2 irAEs were an independent factor that prolonged PFS and OS. Our results are consistent with those of previous studies on patients with other tumor types. A retrospective analysis reported that the development of low-grade irAEs was associated with a significantly better ORR and longer time to next therapy or death in non-melanoma patients treated with ICIs.^10^ In another retrospective analysis of melanoma, patients who experienced grade 3/4 irAEs showed no improvement in ORR compared with those who experienced grade 1/2 irAEs.^11^ In contrast, in a retrospective analysis of HCC, Kennedy et al revealed that the development of grade 3/4 irAEs was associated with a significantly longer OS and PFS and higher ORR than the development of grade 1/2 irAEs and no irAE development.^17^ However, unlike in our study, most patients in their study were treated with ICI monotherapy. Therefore, severe irAEs after treatment with atezolizumab plus bevacizumab may have a different impact on treatment management and prognosis than severe irAEs after ICI monotherapy. In this study, patients with grade 3/4 irAEs had a high rate of permanent discontinuation of treatment despite a high ORR, which may have affected prognosis. Although higher-grade irAEs may indicate a high clinical efficacy due to higher T-cell activation, they may also have a negative impact by causing life-threatening events and treatment discontinuation.^29^ Hence, our results suggest the importance of early recognition and management of irAEs to prevent their progression. However, not all cases can be recognized at earlier grades, and many cases have already progressed to grade 3/4 when symptoms appear. Consequently, more careful management is required to optimize treatment outcomes and prevent harm to patients, regardless of the grade.

A possible bias of this study was the influence of treatment duration, since patients undergoing long-term treatment with ICIs may have an increased likelihood of irAE occurrence due to prolonged drug exposure. Considering the lead time bias, we performed a 9-week landmark analysis. In a real clinical setting, in most cases, the first radiological assessment is performed 6-9 weeks after the start of treatment. Therefore, it is reasonable to select 9 weeks for landmark analysis. Grade 1/2 irAEs were significantly associated with increased PFS (HR, 0.312; 95% CI, 0.143-0.680; *P* = .003) and OS (HR, 0.081; 95% CI, 0.011-0.614; *P* = .015) in the multivariate analysis of the 9-week landmark analysis.

We showed that endocrine irAEs contributed to longer PFS and OS than irAEs of other categories. Endocrine irAEs have been associated with significant improvements in clinical outcome in several previous studies.^30-33^ In addition, endocrine irAEs and other specific types of irAEs have been reported to contribute to favorable prognosis in different malignancies. Freeman et al demonstrated that skin irAEs, including vitiligo, are associated with survival in patients receiving nivolumab for melanoma.^13^ A previous meta-analysis reported that endocrine and dermatologic irAEs were significantly associated with better clinical outcomes in patients with various malignancies.^34^ However, we did not find a correlation between non-endocrine irAEs and prognosis. One possible reason for this is that endocrine irAEs are clinically more manageable and less serious than other irAEs. It is also conceivable that the insufficient number of irAEs due to the small sample size prevented an adequate analysis. Although the mechanisms underlying the association of organ-specific irAEs with the outcome of treatment are unknown, a recent study suggested that the types of irAEs associated with ICI efficacy may have more to do with shared antigens between the tumor and the involved site, rather than any intrinsic association between ICIs and the type of irAEs.^35^ Further research is required to clarify the relationship between the type of irAE and the effect on survival.

This study had some limitations. First, this was a retrospective study, which causes unavoidable bias in patient selection. Second, not all irAEs may have been recognized (especially mild or transient irAEs), and some irAEs are difficult to differentiate from adverse events caused by bevacizumab. Therefore, unrecorded irAEs may have affected the analysis. Third, owing to the small sample size, this study could not examine the relationship between the profile of some irAEs and prognosis. Although only endocrine irAEs were found to be associated with efficacy in this study, an even larger sample size could reveal an association between non-endocrine irAEs and efficacy.

## Conclusion

In conclusion, grade 1/2 irAEs were associated with longer PFS and OS in a real-world population of patients with advanced HCC treated with atezolizumab plus bevacizumab. Grade 3/4 irAEs did not prolong PFS or OS, despite the high response rates. Further studies are required to confirm the potential role of the incidence of irAEs as a predictor of response to ICIs and explore the mechanisms leading to the occurrence of irAEs.

## Data Availability

The data that support the findings of this study are not publicly available because they contain information that could compromise the privacy of the research participants; however, they are available from the corresponding author upon reasonable request.
